# Preclinical alterations in cardiac energetics amongst sarcomere mutation carriers in hypertrophic cardiomyopathy

**DOI:** 10.1186/1532-429X-17-S1-O83

**Published:** 2015-02-03

**Authors:** Rachael Lloyd, Suchi Grover, Susie F Parnham, Pey Wen Lou, Craig Bradbrook, Laura Yeates, Gemma Correnti, Eric Haan, John J Atherton, Christopher Semsarian, Joseph Selvanayagam

**Affiliations:** 1 Flinders Medical Centre, Carlton South, VIC, Australia; 2 South Australian Health and Medical Research Institute (SAHMRI), Adelaide, SA, Australia; 3 Flinders University, Adelaide, SA, Australia; 4 Royal Brisbane Hospital, Brisbane, QLD, Australia; 5 Agnes Ginges Centre for Molecular Cardiology, Centenary Institute, Sydney, NSW, Australia; 6 SA Clinical Genetics Service, Adelaide, SA, Australia

## Background

Hypertrophic cardiomyopathy (HCM) is characterised by reduced myocardial tissue oxygenation (assessed using blood oxygen level dependent (BOLD) CMR imaging) during stress, as well as reduced myocardial perfusion reserve (MPRI) due to coronary microvascular dysfunction. In HCM gene carriers without the HCM phenotype, it has been suggested that only oxygenation is impaired. [1] It remains unclear whether this relates to early cardiac remodelling/ diastolic dysfunction, or whether oxygen consumption is intrinsically altered with sarcomere mutations. We sought to assess the BOLD signal change during vasodilator stress in a homogenous group of MYPBC3 positive HCM patients (some with clinical HCM, and some with no phenotypic features of HCM), and normal controls.

## Methods

A total of 21 subjects with MYPBC3 mutations (11 with (G+P+) and 10 without (G+P-) phenotypic evidence of HCM on MRI or echocardiogram), and 6 normal controls underwent CMR scanning at 3.0 T. Myocardial function, as well as oxygenation (BOLD signal intensity change) during rest and adenosine stress was performed.

All HCM (both G+ P+ and G+P-) patients had transthoracic echocardiography performed, including evaluation of diastolic function using pulsed-wave and tissue Doppler.

## Results

Mean age (yrs) was similar across the groups (40 for G+P+, 39.5 for G+P- and 45 for normal controls). Maximal septal thickness was 21mm in the G+P+ group, vs 9.2 for the G+P- group. As expected, the G+P+ group had a blunted oxygenation response during stress (BOLD SI change -4.8 ± 10.3% vs controls 11.1 ± 8.1, p= 0.003). The G+P- group also showed reduced oxygen response during stress (BOLD SI change 2.5 ± 4.8% vs controls, p= 0.009). 8 of the 11 (73%) G+P+ HCM patients had diastolic dysfunction, vs none of the G+P- group. Mean E/E' was 13.3 ± 2.8 for the G+P+ group vs 8 ± 1.9 for the G+P- group, p= <.00001.

## Conclusions

HCM gene carriers without hypertrophy show reduced myocardial oxygenation during vasodilator stress compared to normal controls. Our findings support the hypothesis that alterations in cardiac energetics may be due to the presence of sarcomere mutations rather than cardiac remodelling or diastolic dysfunction which occur at a later stage.

## Funding

National Heart Foundation of Australia.

## References

[B1] Karamitsos Theodoros D Dass Sairia Suttie Joseph Sever Emily Birks Jacqueline Holloway Cameron J Robson Matthew D Jerosch-Herold Michael Watkins Hugh Neubauer Stefan Blunted Myocardial Oxygenation Response During Vasodilator Stress in Patients With Hypertrophic Cardiomyopathy Oxford, United Kingdom; and Boston, Massachusetts 10.1016/j.jacc.2012.12.024 PMC3595528 23498131

